# Global DNA methylation profiling reveals chromosomal instability in IDH-mutant astrocytomas

**DOI:** 10.1186/s40478-022-01339-2

**Published:** 2022-03-09

**Authors:** Yan Liu, Adwait Amod Sathe, Kalil G. Abdullah, Samuel K. McBrayer, Steven H. Adams, Andrew J. Brenner, Kimmo J. Hatanpaa, Mariano S. Viapiano, Chao Xing, Jamie M. Walker, Timothy E. Richardson

**Affiliations:** 1 Eugene McDermott Center for Human Growth & Development, University of Texas Southwestern Medical Center, Dallas, TX 75390 USA; 2 Department of Neurosurgery, University of Pittsburgh School of Medicine, 200 Lothrop St, Pittsburgh, PA 15213 USA; 3 Hillman Comprehensive Cancer Center, University of Pittsburgh Medical Center, 5115 Centre Ave, Pittsburgh, PA 15232 USA; 4 Simmons Comprehensive Cancer Center, University of Texas Southwestern Medical Center, Dallas, TX 75390 USA; 5 Children’s Medical Center Research Institute, University of Texas Southwestern Medical Center, Dallas, TX 75390 USA; 6 Department of Pathology, Stony Brook University Hospital, Stony Brook, NY 11794 USA; 7 Department of Internal Medicine, Division of Hematology & Oncology, University of Texas Health San Antonio, San Antonio, TX 78229 USA; 8 Mays Cancer Center, University of Texas Health San Antonio, San Antonio, TX 78229 USA; 9 Department of Pathology, University of Texas Southwestern Medical Center, Dallas, TX 75390 USA; 10 Department of Neuroscience and Physiology, State University of New York, Upstate Medical University, Syracuse, NY 13210 USA; 11 Department of Neurosurgery, State University of New York, Upstate Medical University, Syracuse, NY 13210 USA; 12 Department of Bioinformatics, University of Texas Southwestern Medical Center, Dallas, TX 75390 USA; 13 Department of Population and Data Sciences, University of Texas Southwestern Medical Center, Dallas, TX 75390 USA; 14 Department of Pathology and Laboratory Medicine, Glenn Biggs Institute for Alzheimer’s & Neurodegenerative Disease, University of Texas Health San Antonio, 7703 Floyd Curl Dr., MC 8070, San Antonio, TX 78229 USA

**Keywords:** Glioma, Astrocytoma, Glioblastoma, Methylation profiling, Chromosomal instability, Copy number variation, IDH-mutation

## Abstract

Diffusely infiltrating gliomas are among the most common central nervous system tumors in adults. Over the past decade, the subcategorization of these tumors has changed to include both traditional histologic features and more recently identified molecular factors. However, one molecular feature that has yet to be integrated is the presence/absence of chromosomal instability (CIN). Herein, we use global methylation profiling to evaluate a reference cohort of IDH-mutant astrocytomas with and without prior evidence of CIN (n = 42), and apply the resulting methylation-based characteristics to a larger test cohort of publicly-available IDH-mutant astrocytomas (n = 245). We demonstrate that IDH-mutant astrocytomas with evidence of CIN cluster separately from their chromosomally-stable counterparts. CIN cases were associated with higher initial histologic grade, altered expression patterns of genes related to CIN in other cancers, elevated initial total copy number burden, and significantly worse progression-free and overall survival. In addition, in a grade-for-grade analysis, patients with CIN-positive WHO grade 2 and 3 tumors had significantly worse survival. These results suggest that global methylation profiling can be used to discriminate between chromosomally stable and unstable IDH-mutant astrocytomas, and may therefore provide a reliable and cost-effective method for identifying gliomas with chromosomal instability and resultant poor clinical outcome.

**Supplementary Information:**

The online version contains supplementary material available at 10.1186/s40478-022-01339-2.

## Introduction

With the recent introduction of the 2021 5th Edition of the WHO Classification of Tumours of the Central Nervous System, diffuse gliomas in adults are now defined by both histologic and molecular features, and in many cases molecular features outrank traditional histologic classification in both diagnosis and grade [18]. Oligodendrogliomas (WHO grades 2–3) are now defined by the presence of simultaneous deletion of chromosomal arms 1p and 19q, as well as mutation in either *IDH1* or *IDH2*. Moreover, what was previously classified by histology as “astrocytoma” and “glioblastoma” has now been divided into IDH-mutant astrocytoma (WHO grades 2–4) and IDH-wildtype glioblastoma (WHO grade 4). Notably, IDH-mutant astrocytomas with homozygous *CDKN2A* deletion are now considered WHO grade 4 regardless of histologic features [2, 18], although numerous other mutations, specific copy number variants, and other molecular features have been suggested as relevant to prognosis and potentially tumor grade [21].

One potentially useful molecular feature that may help explain the underlying heterogeneity in clinical outcome between IDH-mutant astrocytomas of the same WHO grade is chromosomal instability (CIN). Previously, we have demonstrated that overall copy number variation (CNV), distributed across the entire genome, tends to increase with histologic grade in IDH-mutant astrocytomas [28], and IDH-mutant grade 2–3 astrocytomas with poor clinical outcomes have incongruously elevated overall CNV at the time of initial diagnosis [27, 29]. This high CNV occurs frequently in cases with other aggressive molecular features including *CDK4* amplification and homozygous *CDKN2A* deletion, although in many cases elevated CNV is the only molecular factor present to suggest poor prognosis [22, 27, 29]. These IDH-mutant astrocytomas can be stratified solely by CNV level with thresholds of approximately 310–465 megabase pairs (Mbp) (~ 10–15% of the total genome) separating patient outcomes [23, 28, 32]. Additionally, CNS and other solid tumors with previously identified CIN [35] can reliably be identified based on mRNA profiles of specific gene sets (CIN25, CIN70) in cases with available whole exome sequencing [6, 28], although this method can add significant processing time and costs. There is a wealth of evidence suggesting that CIN is a contributing factor to the aggressiveness of a subset of otherwise lower-grade IDH-mutant astrocytomas, however because CIN is a dynamic and ongoing process and a single biopsy or resection specimen represents only a snapshot of the temporal and spatial molecular evolution of the neoplasm, detection may be difficult and impractical in many clinical settings [35, 36].

Global DNA methylation profiling is a technique that has become relatively common in the past 5 years to diagnose and categorize CNS neoplasms (as well as identify new entities) based on epigenetic features [5, 24]. In this report, we leverage global methylation profiling to distinguish CIN and chromosomal stable (CS) phenotypes in IDH-mutant astrocytomas. We utilized a small cohort of IDH-mutant astrocytomas (n = 42) with known CIN or CS status to identify epigenetic differences between the two groups, and then applied this method using an unbiased approach to a larger cohort of publicly-available IDH-mutant astrocytomas (n = 245) to verify these epigenetic signatures and investigate the clinical and molecular differences between cases clustering as CIN or CS by methylation profiling.

## Methods

### Case selection

Reference cohort: We analyzed 42 IDH-mutant astrocytomas (2021 WHO grades 2–4) from previous studies with multiple previously established lines of evidence suggesting either chromosomal instability (CIN) or chromosomal stability (CS) status, including mRNA profiling of gene panels linked to CIN (CIN70) [6, 28], evidence of cell-to-cell chromosomal heterogeneity [29], mutations in genes with known roles in the maintenance of chromosomal stability [22, 27, 28], copy number profile evidence of significant spatial chromosomal heterogeneity [19], and significantly elevated CNV with multiple biopsies/resections reflective of rapid chromosomal alteration (Additional File 1: Fig. 1 ). All cases represent the first resection specimen, before treatment was initiated, and were classified according to WHO 2021 integrated histologic/molecular criteria [18].

Test cohort: Using the cBioPortal interface [8, 14], we performed a search of IDH-mutant astrocytoma (WHO grade 2–4) from publicly-available datasets [1, 3, 4, 7]. The original histologic diagnoses reported included astrocytoma, anaplastic astrocytoma, oligodendroglioma, anaplastic oligodendroglioma, oligoastrocytoma, anaplastic oligoastrocytoma, and glioblastoma. All cases were reclassified according to WHO 2021 integrated histologic/molecular criteria using *IDH1/2*, 1p/19q, *TP53*, and *ATRX* status [18]. All cases included in this study represented the first resection specimen. From these large datasets, a total of 245 cases meeting the criteria for IDH-mutant astrocytoma (WHO grade 2–4) with available global methylation profiling, whole exome sequencing, mRNA expression levels, and copy number variation (CNV) were selected for further analysis.

### Genetic and epigenetic analysis

As previously detailed [13, 22, 27, 28], DNA methylation data (Illumina Human Methylation 450) and gene expression data (Illumina HiSeq, RNASeq) were downloaded and analyzed with R 3.4.1 (http://www.R-project.org/), TCGAbiolinks (https://bioconductor.org/packages/release/bioc/html/TCGAbiolinks.html), and Qiagen’s IPA tool (www.qiagen.com/ingenuity) (Qiagen, Hilden, Germany) [10, 33]. The Affymetrix SNP 6.0 microarray data normalized to germline for copy number analysis for the same publicly-available cases was downloaded from Broad GDAC Firehose (http://gdac.broadinstitute.org/runs/stddata__2016_01_28/).

Molecular subtype classification was performed utilizing the cloud-based DNA methylation classifier (www.molecularneuropathology.org) [5]. Unsupervised hierarchical clustering of the most differently expressed DNA methylation regions was performed on each reference cohort case using Euclidian distance measures, Pearson correlation coefficient and average linkage, and then repeated in the larger test cohort [19, 34]. Heatmaps were generated using ComplexHeatmap (https://bioconductor.org/packages/release/bioc/html/ComplexHeatmap.html). Uniform Manifold Approximation and Projection (UMAP) [20] and t-Distributed Stochastic Neighbor Embedding (t-SNE) [5] plotting were performed with R umap and tsne packages, using the same distance metrics and default parameters. *MGMT* promoter methylation was reported in the majority of cases in cBioPortal and confirmed with methylation array data. For CIN70 RNAseq data, normalized gene expression level ranking was performed as previously described [28].

The fraction of CNV was calculated from data in cBioPortal as the fraction of the genome (expressed as percent of nucleotide base pairs) with log2 of copy number > 0.3 (gain or loss) following the procedure described by Gao et al. and visualized with the cBioPortal interface [14]. CNV was quantified by percentage of the total genome, as previously described [13, 22, 27]. Mutation analysis was performed on whole exome sequencing and included all recurrent mutations as well as genes with previously identified roles in cell proliferation, cancer and malignant progression in CNS neoplasms and other cancers, and maintenance of chromosomal stability [22, 27]. Gene variant annotation was performed using The 1000 Genomes Project, COSMIC, dbSNP, ClinVar, CanProVar 2.0, and FATHMM-MKL [12, 15, 16, 30, 31, 37].

### Statistical analysis

Differences in patient age at diagnosis and CNV were evaluated using student’s t-test. The significance of Kaplan–Meier survival curves was calculated using the Mantel-Cox test (Log-rank test). Proportion of cases with mutations specifically associated with genome instability, as well as gender, CIN70, *MGMT* promoter methylation, and 2016/2021 WHO grade were calculated using Fisher’s Exact test. All statistical calculations were performed with GraphPad Prism version 9 (GraphPad, La Jolla, CA).

## Results

### Methylation profile, molecular features, and clinical characteristics of reference cohort

**Fig. 1:**
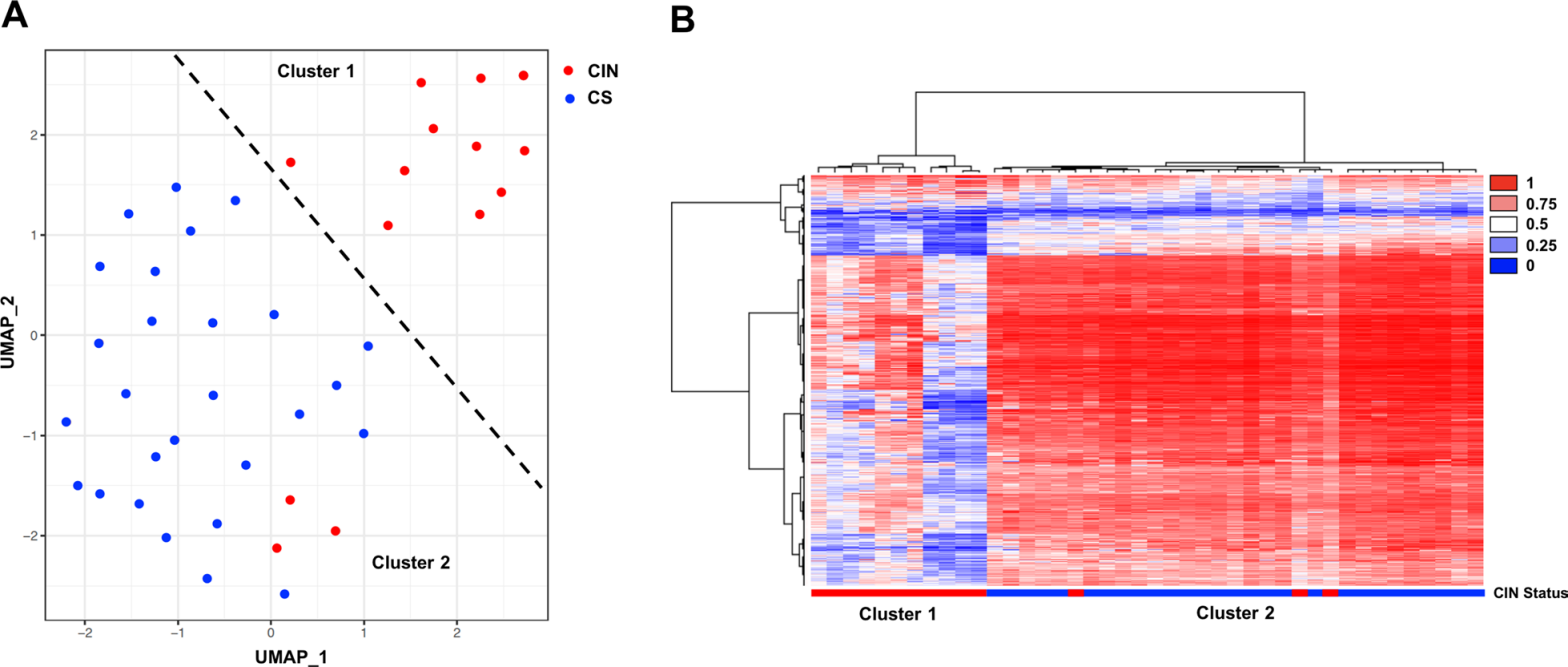
**a** Uniform manifold approximation and projection (UMAP) plotting demonstrating separate clustering between reference cohort WHO grade 2–4 IDH-mutant astrocytomas with chromosomal instability (CIN) and those with chromosomal stability (CS), and **b** cluster analysis heatmap summarizing DNA methylation profiles of the reference cohort (n = 42)

Methylation profiling was performed on 14 IDH-mutant astrocytomas with previously established evidence of chromosomal instability (CIN) [19, 22, 28, 29] and 28 “chromosomally stable” (CS) cases (Fig. 1), demonstrating two distinct methylation clusters with UMAP analysis: cluster 1, comprising 11 of the previously established CIN cases, and cluster 2, comprising all 28 CS cases and 3 CIN cases (78.6% sensitivity, 100% specificity, 100% positive predictive value (PPV), 90.3% negative predictive value (NPV)), suggesting that there may be epigenetic differences which can be determined by analysis of methylation sites. UMAP and t-SNE analysis demonstrated statistically equivalent results.

Compared to cluster 2, cluster 1 had significantly worse progression-free survival (PFS; 31.1 vs 95.1 months; *p* = 0.0077) and overall survival (OS; 36.8 vs 122.4 months; *p* < 0.0001) (Table 1). In addition, cluster 1 cases also had significantly higher overall copy number variation (CNV) compared to cluster 2 (20.2 ± 5.8% vs 7.0 ± 1.0%; *p* = 0.0018), significantly more frequent cases with high CIN70 mRNA expression levels (*p* < 0.0001), a set of 70 genes with tight correlation between high expression and the presence of CIN [6, 28], higher 2016 WHO grade (*p* = 0.0037), and higher 2021 WHO grade (*p* = 0.0224). No significant differences were identified in terms of patient gender, age at diagnosis, *MGMT* promoter methylation status, or other mutations or copy number changes.

Of particular interest are the three cases with known CIN that clustered with CS cases (Fig. 1a). All three cases demonstrated *MGMT* promoter methylation, and were 2021 WHO grade 2, 3, and 4. No difference in age was identified between these three cases and cluster 1 or the other cluster 2 cases. These cases have significantly higher overall CNV (15.3% ± 8.1%) compared to the other cluster 2 cases (*p* = 0.0285), but statistically equivalent CNV to cluster 1 (*p* = 0.6845). No significant differences were noted in PFS or OS between these three cases and cluster 1 or cluster 2, although there was a non-significant trend toward shorter PFS and OS in these cases compared to cluster 2 and longer PFS and OS compared to cluster 1.

### Methylation profile, molecular features, and clinical characteristics of test cohort

**Fig. 2:**
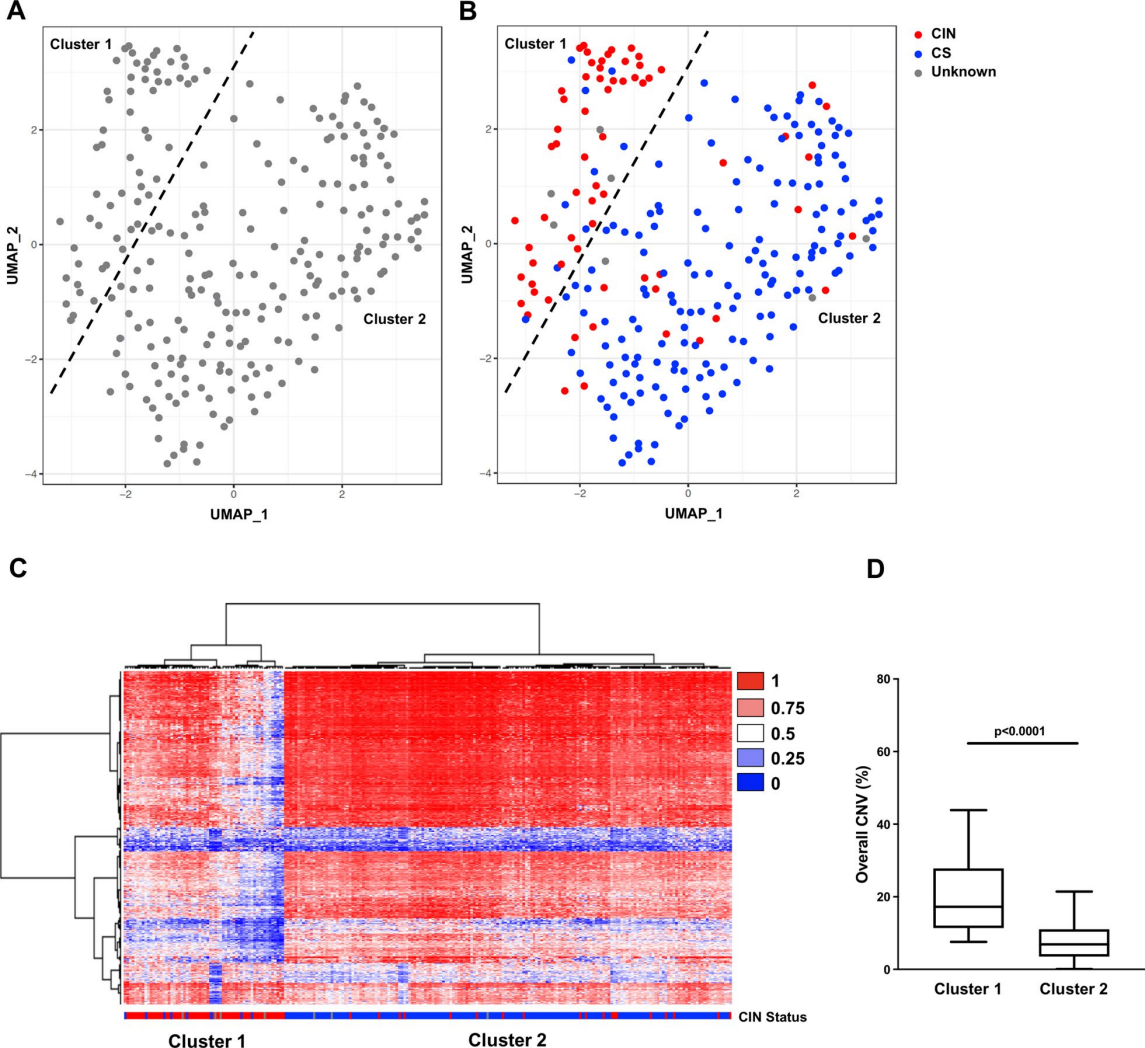
**a–b** Uniform manifold approximation and projection (UMAP) plotting demonstrating separate clustering between the test cohort WHO grade 2–4 IDH-mutant astrocytomas with chromosomal instability (CIN) and those with chromosomal stability (CS). **c** Cluster analysis heatmap summarizing DNA methylation profiles of the test cohort (n = 245). **d** Cases in cluster 1 (CIN) demonstrated significantly higher overall copy number variation on initial resection compared to cases in cluster 2 (CS)

Methylation profiling was performed with UMAP analysis on 245 IDH-mutant astrocytomas, and identified 57 cases (23.3%) with a similar pattern of methylation probe levels to cluster 1 of the reference cohort (CIN) and 188 cases with similar methylome characteristics to cluster 2 of the reference cohort (CS) (Fig. 2a–c). UMAP plotting demonstrated that all reference cohort cases aligned with their respective test cohort clusters. UMAP and t-SNE plotting methods demonstrated statistically equivalent results.

Cluster 1 cases had an elevated level of CNV at initial biopsy (21.2 ± 1.9% vs 7.4 ± 0.5%; *p* < 0.0001) (Fig. 2d & Table 1), distributed across the entire genome [22, 27], as well as a higher percentage of cases with “high” CIN70 expression levels (75.5% vs 8.7%; *p* < 0.0001), compared to their cluster 2 counterparts (Table 1). Cases in cluster 1 also had significantly more frequent mutations in genes with known functions associated with maintaining overall genome stability [22, 27, 28] compared to cluster 2 cases (17.5% vs 7.9%; *p* = 0.0426). Overall, 81.4% of cases in cluster 1 were positive for CIN by at least one additional method compared to 12.7% of cases in cluster 2 (*p* = 0.0001; 65.7% sensitivity, 94.7% specificity, 83.0% PPV, 87.6% NPV) (Fig. 2b). Of note, definitive CIN/CS status could not be determined in 7 cases (4 cluster 1 and 3 cluster 2 cases). Cluster 1 cases also tended to initially present at higher 2021 WHO grades (*p* = 0.0007) with no significant difference in any other molecular alteration, including *MGMT* promoter methylation status (Table 1).

**Table 1:** Clinical and molecular features in IDH-mutant CIN and CS astrocytomas

Cluster	n	Gender (M:F)	Age (years)	WHO Grade		CNV (%)	CIN70 mRNA expression (High:Low)	*MGMT* promoter methylation status	Median PFS (months)	Median OS (months)
				2016 (2:3:4)	2021 (2:3:4)					
*Reference cohort*										
Cluster 1 (CIN)	11	4:7	38.4 ± 2.9	2:8:1	1:6:4	20.2 ± 5.8	11:0	7:4	31.1	36.8
Cluster 2 (CS)	31	19:12	39.0 ± 1.9	21:10:0	17:9:5	7.0 ± 1.0	3:27	10:20	95.1	122.4
p-value	–	0.1795	0.7779	**0.0037**	**0.0224**	**0.0018**	** < 0.0001**	0.1509	**0.0077**	** < 0.0001**
*Test cohort*										
Cluster 1 (CIN)	57	26:31	41.9 ± 1.6	17:36:4	15:26:16	21.2 ± 1.9	37:12	46:11	38.0	50.5
Cluster 2 (CS)	188	110:78	37.4 ± 1.5	106:76:6	95:72:21	7.4 ± 0.5	16:167	162:26	62.0	98.2
p-value	–	0.0956	0.0756	**0.0010**	**0.0007**	** < 0.0001**	** < 0.0001**	0.3000	**0.0034**	**0.0002**

*Note* CNV copy number variation, *CIN* chromosomal instability, *CS* chromosomally stable, *PFS* progression-free survival, *OS* overall survival; bold = significant to a level of 0.05

### Methylation profiling of CIN as a prognostic factor

**Fig. 3:**
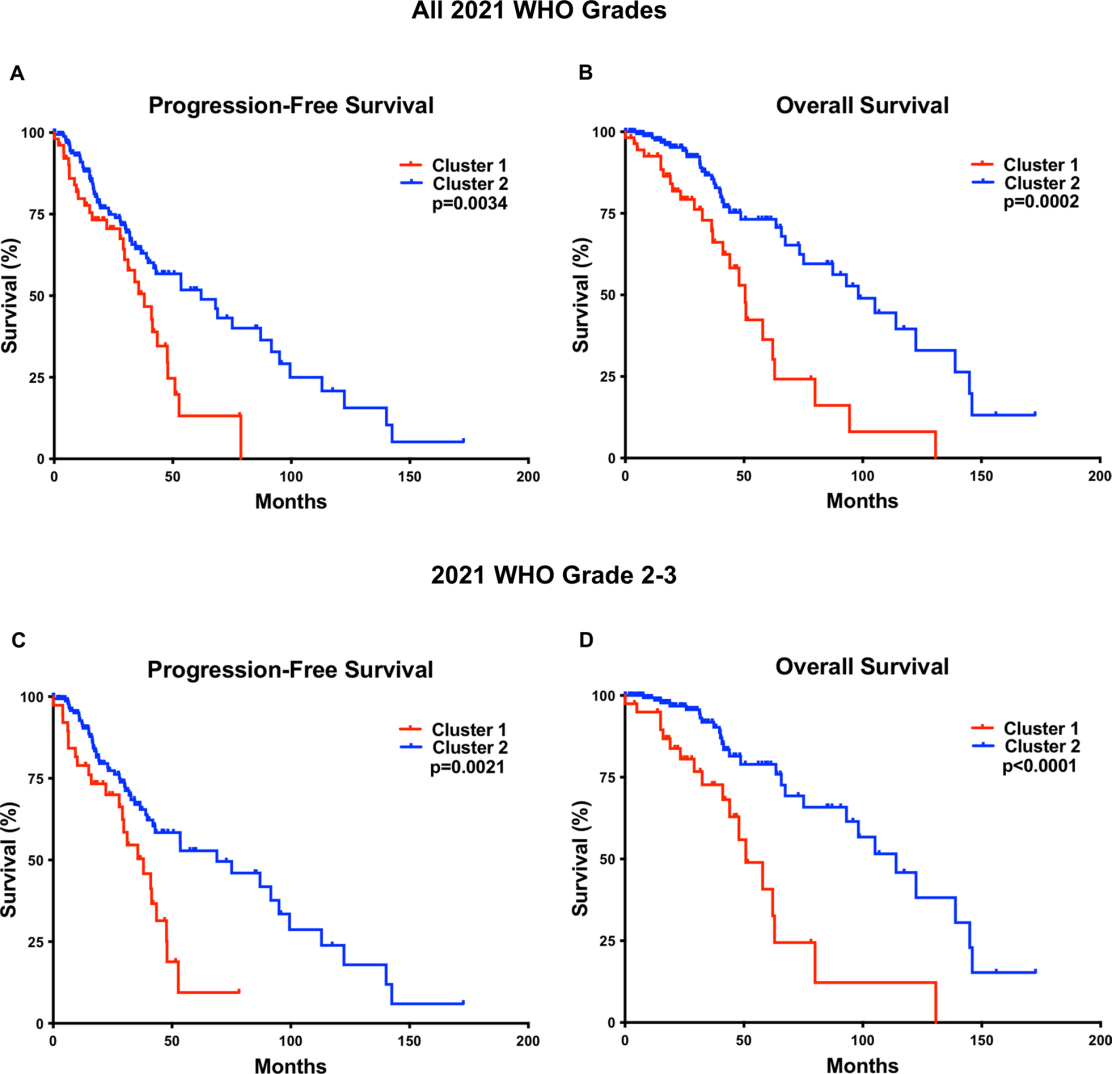
Combined 2021 WHO grade 2–4 cases from the test cohort demonstrating worse progression-free survival (PFS) **a** and overall survival (OS) **b** in cluster 1 compared to cluster 2. 2021 WHO grade 2–3 combined cases (“lower-grade astrocytoma”) from cluster 1 had worse PFS **c** and OS **d** compared to grade 2–3 cases from cluster 2

In the test cohort, cluster 1 had significantly worse PFS (38.0 vs 62.0 months; *p* = 0.0034) (Fig. 3a) and OS (50.5 months vs 98.2 months; *p* = 0.0002) (Fig. 3b), compared to cluster 2. Stratifying cases by these methylation profiles yielded results comparable to stratifying by current 2021 WHO grade (Additional File 2: Fig. 2), overall CNV threshold of 10% or 15% [23, 28], and CIN70 mRNA expression levels [28].

**Fig. 4.**
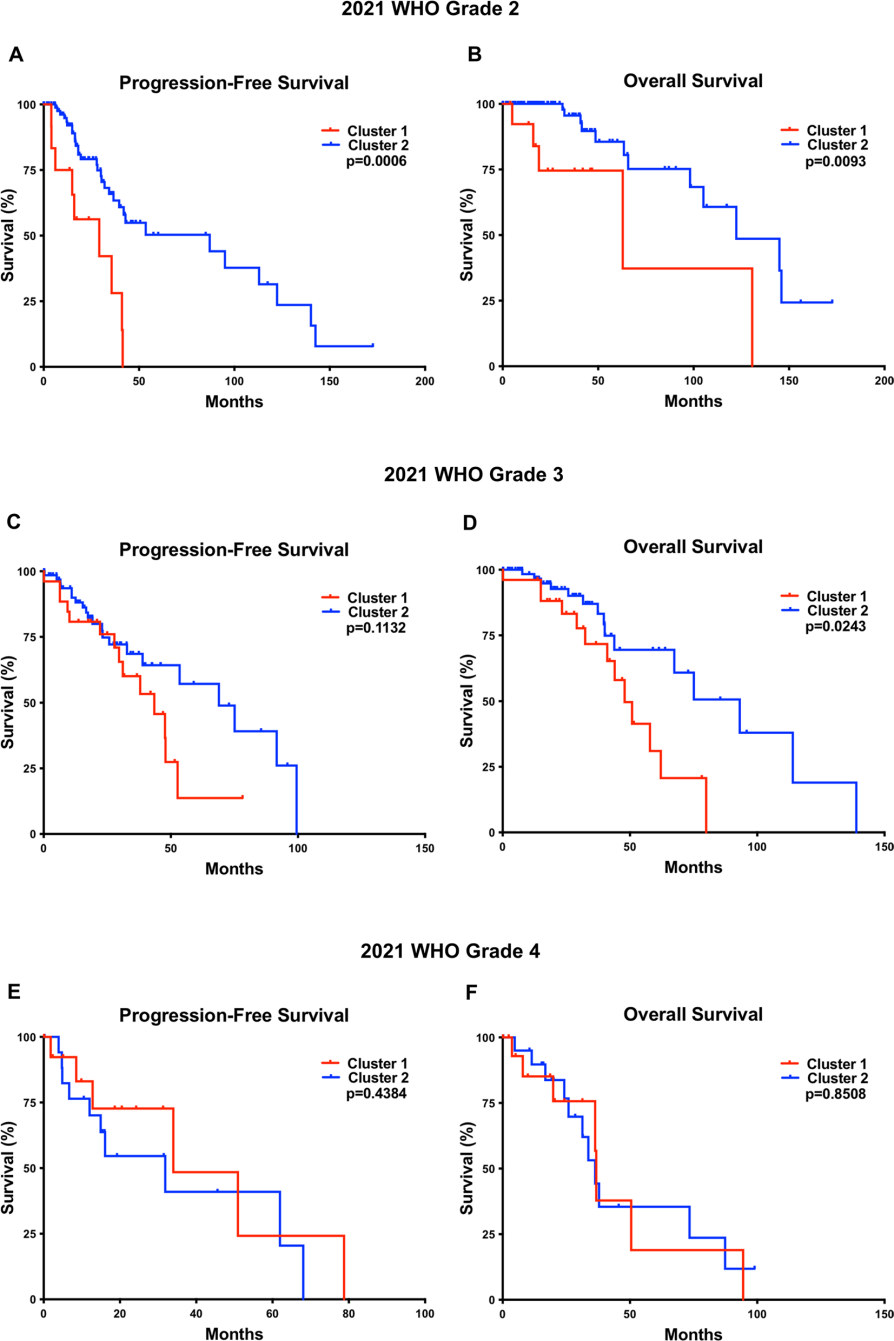
2021 WHO grade 2 cases from cluster 1 had worse progression-free survival (PFS) **a** and overall survival (OS) **b** compared to grade 2 cases from cluster 2. No significant difference was found between 2021 WHO grade 3 cases from cluster 1 and 2 in terms of PFS **c**, however grade 3 cases from cluster 1 had significantly worse OS compared to grade 3 cases from cluster 2 **d**. No significant difference was found between 2021 WHO grade 4 cases from cluster 1 and 2 in terms of PFS **e** or OS **f**

“Lower-grade astrocytomas” (WHO grade 2–3) demonstrated a significant difference in overall CNV (22.0 ± 2.4% vs 7.8 ± 1.8; *p* = 0.0003), patient age at diagnosis (43.3 ± 2.1 vs 36.9 ± 1.3 years; *p* = 0.0245), PFS (38.0 vs 68.9 months; *p* = 0.0021) and OS (50.8 vs 114; *p* < 0.0001) in the cluster 1 cases (n = 41) compared to the cluster 2 cases (n = 167) (Fig. 3c, d and Table 2). In a grade-for-grade analysis (Fig. 4), WHO grade 2 cluster 1 cases had significantly worse PFS (29.1 vs 87.1 months; *p* = 0.0006) and OS (62.9 vs 122.4 months; p = 0.0093), compared to WHO grade 2 cluster 2 cases. WHO grade 3 cluster 1 cases had significantly worse OS (47.9 vs 93.1 months; *p* = 0.0243) compared to WHO grade 3 cluster 2 cases, however no significant difference was identified between the two WHO grade 3 clusters in terms of PFS (*p* = 0.1132), and no significant difference was identified in terms of PFS (*p* = 0.4384) or OS (*p* = 0.8505) in the WHO grade 4 tumors. Additionally, WHO grade 2, 3, and 4 IDH-mutant astrocytomas in cluster 1 had significantly higher CIN compared to their grade-matched cluster 2 counterparts, but no other significant clinical or molecular differences (Table 2).

## Discussion

Diffuse gliomas in adults remain a largely incurable disease [21, 25]. However, more recently identified molecular features and epigenetic classification systems have helped to refine the diagnostic criteria beyond traditionally used histologic features, as well as identify novel, potentially targetable genetic alterations, some of which have now been codified into official diagnostic algorithms as the new standard of care [18, 26]. Still, there is significant variation in terms of individual patient morbidity and mortality that is partially unaccounted for by recent changes to the WHO diagnostic and grading system.

Chromosomal instability (CIN) is a known mechanism for malignant progression in many systemic cancers. This process results in large scale and rapid alterations to whole chromosomes or chromosomal regions that may affect numerous genes and cell processes and may cause significant intratumoral and cell-to-cell molecular heterogeneity, producing clones that may vary in malignancy, tendency to invade surrounding tissue, ability to evade immune regulation, and/or resistance to traditional therapies [36]. While this process is relatively well known and the mechanisms underlying it have long been studied, detecting the presence of CIN from a single biopsy or resection specimen may be challenging and impractical to perform from a technical standpoint, and a number of direct and indirect methods for CIN detection have been proposed [35]. Additionally, while CIN frequently results in more aggressive behavior in cancer, the process itself may be targeted by a number of therapeutic agents that are currently FDA-approved or in clinical trials for other cancer types [35], raising the possibility that these drugs may be useful as adjuvant therapies in diffuse gliomas with CIN. While there are few studies that investigate the effect of CIN and overall CNV in diffuse gliomas, we and others have used large publicly-available and institutional cohorts of IDH-mutant astrocytoma and IDH-wildtype glioblastoma to demonstrate that CIN can be readily identified through whole genome copy number profiling and whole exome sequencing in certain subsets. Importantly, stratification of cases using these methods has as good or better predictive power as previously codified histologic or molecular features [22, 23, 27–29, 32].

In this report, we analyzed a small cohort of IDH-mutant astrocytomas with known CIN or CS status, determined by multiple distinct methods, to determine if this one characteristic would yield separate methylation profile clusters (Fig. 1). We then analyzed a larger cohort to verify that the identified methylome characteristics and resulting clustering pattern would be corroborated in a blindly evaluated cohort (Fig. 2). Our findings demonstrate that methylation profiling can distinguish IDH-mutant astrocytomas with and without CIN: cluster 1 had a distinct methylation profile, significantly greater proportion of cases with at least one measure of CIN, significantly higher CNV levels at the time of initial surgery, and significantly worse PFS and OS compared to cluster 2 (Table 1). It is worth noting that cluster 1 cases tended to be higher grade at first diagnosis, which perhaps is to be expected since higher grades tended to be associated with more genomic instability [9, 28]. On the other hand, cases with CIN may present at higher histologic grades simply because they progress more rapidly and so on average may be identified later in their evolution [29]. However, methylation profiling identified 15 grade 2 cases and 26 grade 3 cases in the CIN cluster, suggesting that methylation profiling is a potentially viable method for identifying cases with CIN and resulting poor prognosis even in these lower-grade astrocytomas (Table 2).

**Table 2:** Clinical and molecular features in test cohort IDH-mutant CIN and CS astrocytomas by grade

2021 WHO Grade	n	Gender (M:F)	Age (years)	CNV (%)	*MGMT* promoter methylation	Median PFS (months)	Median OS (months)
*Lower-Grade (2–3)*							
Cluster 1 (CIN)	41	19:22	43.3 ± 2.1	22.0 ± 2.4	32:9	38.0	50.8
Cluster 2 (CS)	167	97:70	36.9 ± 1.3	7.8 ± 1.8	144:39	68.9	114
p-value	–	0.2194	**0.0245**	**0.0003**	0.9281	**0.0021**	** < 0.0001**
*Grade 2*							
Cluster 1 (CIN)	15	5:10	46.8 ± 3.7	17.0 ± 2.2	8:7	29.1	62.9
Cluster 2 (CS)	95	54:41	36.2 ± 2.9	7.0 ± 0.5	82:29	87.1	122.4
p-value	–	0.1028	0.1585	** < 0.0001**	0.1279	**0.0006**	**0.0093**
*Grade 3*							
Cluster 1 (CIN)	26	14:12	41.9 ± 2.5	24.1 ± 3.3	24:2	43.5	47.9
Cluster 2 (CS)	72	43:29	37.9 ± 1.4	8.7 ± 0.8	62:10	68.9	93.1
p-value	–	0.6474	0.1521	** < 0.0001**	0.5073	0.1132	**0.0243**
*Grade 4*							
Cluster 1 (CIN)	16	7:9	38.7 ± 1.9	19.4 ± 2.5	14:2	34.0	36.8
Cluster 2 (CS)	21	13:8	40.9 ± 1.6	14.0 ± 1.3	18:3	31.9	36.3
p-value	–	0.3309	0.3795	**0.0481**	0.8749	0.4384	0.8508

*Note* CNV copy number variation, *CIN* chromosomal instability, *CS* chromosomally stable, *PFS* progression-free survival, *OS* overall survival, bold = significant to a level of 0.05

This report helps to further solidify the concept that CIN and CNV are features that can be measured and used as prognostic factors in subsets of IDH-mutant astrocytomas in certain situations. The fact that the CIN cluster cuts across 2021 WHO grades and the fact that the CIN cluster has significantly elevated overall CNV, even in grade 2 tumors, supports the idea that CIN is present in a subset of these tumors and may be detected before histologic indicators of tumor progression or aggressive behavior are present, and suggests that CIN is present early in tumor development, representing a fundamentally altered biology compared to IDH-mutant astrocytomas without this feature. Incongruously elevated CNV identified in newly diagnosed lower-grade IDH-mutant astrocytomas likely results from underlying CIN and may be a driver of poor clinical behavior and outcome, rather than simply being a reflection of other molecular processes.

These data represent only a small sample of IDH-mutant astrocytomas with methylation profiling paired with other molecular and clinical data and as such does not necessarily define the full molecular parameters of these tumors. Nonetheless, our findings do serve as a proof-of-concept that methylation profiling, an already widely accepted modality for diagnosing CNS neoplasms, may be employed to identify this underlying characteristic and separate out cases with CIN which may have more aggressive clinical courses. Given the role that global methylation profiling is beginning to play in clinical practice, this may be an ideal way to routinely screen for characteristics such as CIN, rather than relying on more intensive and less practical methods, such as single cell sequencing to identify the minority of cases in which CIN may be present [35]. Still, more work is needed to further define the epigenetic signature associated with CIN in much larger and more diverse cohorts of IDH-mutant astrocytoma, as well as to place these CIN-positive diffuse gliomas in the greater context of all CNS neoplasms by methylome profiling [24]. It is important to rapidly identify genetic and epigenetic characteristics that affect diagnosis and prognosis to ensure appropriate treatment, and multiple studies have recently shown that this type of in-depth molecular data can be available in time to guide neurooncological treatment [11, 17, 26].

In conclusion, these data further support the idea that the presence of CIN and elevated CNV levels are crucial data points in predicting clinical outcome, and may one day be useful targets for personalized therapies. Methylation profiling has proven to be a powerful tool for CNS neoplasm diagnosis and may potentially identify IDH-mutant astrocytomas with underlying CIN, even if histologically consistent with 2021 WHO grade 2 or 3, to help further refine prognosis and improve therapeutic design in the future.

## Supplementary Information

**Additional file 1. Fig. 1** Copy number variation differences between initial biopsies and tumor recurrences in IDH-mutant and IDH-wildtype astrocytomas (A) and IDH-mutant astrocytomas with known CIN and CS status (B)

**Additional file 2. Fig. 2** Kaplan-Meier analysis of the full test cohort demonstrating significant differences in progression-free (A) and overall survival (B) between cases stratified by 2021 WHO grade

**Additional file 3. Table 1** List of IDH-mutant astrocytoma cases

## Data Availability

Data used in this study is available at www.cbioportal.org, https://portal.gdc.cancer.gov/, and https://www.cancer.gov/about-nci/organization/ccg/research/structural-genomics/tcga. The cases used in this study are included as Additional File 3: Table 1.
