# Supplementary figures and images for: Global DNA methylation profiling reveals chromosomal instability in IDH-mutant astrocytomas
Source: Acta Neuropathol Commun. 2022 Mar 9;10:32. doi: 10.1186/s40478-022-01339-2 (PMC8908645; doi:10.1186/s40478-022-01339-2)

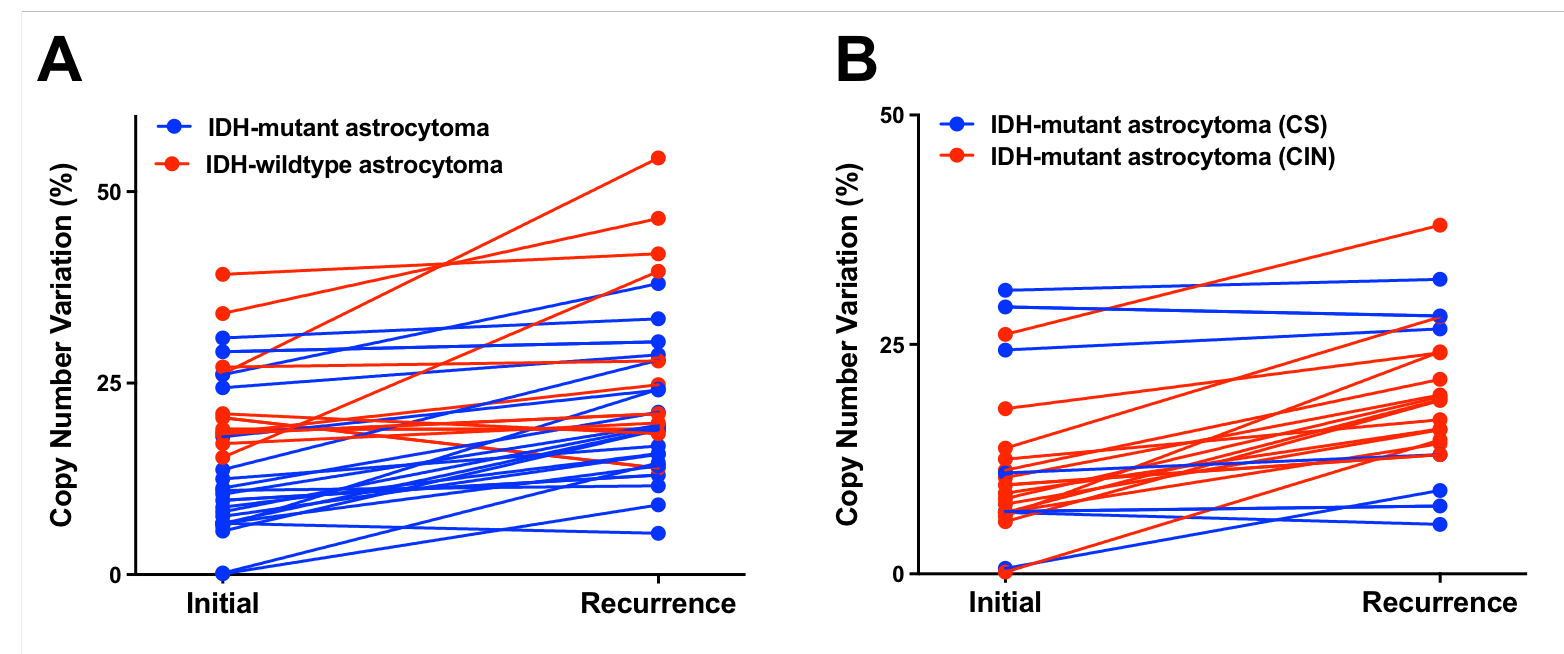

Supplement: Supplementary file 1 — Additional file 1. Fig. 1 Copy number variation differences between initial biopsies and tumor recurrences in IDH-mutant and IDH-wildtype astrocytomas (A) and IDH-mutant astrocytomas with known CIN and CS status (B) [file 40478_2022_1339_MOESM1_ESM.tiff]

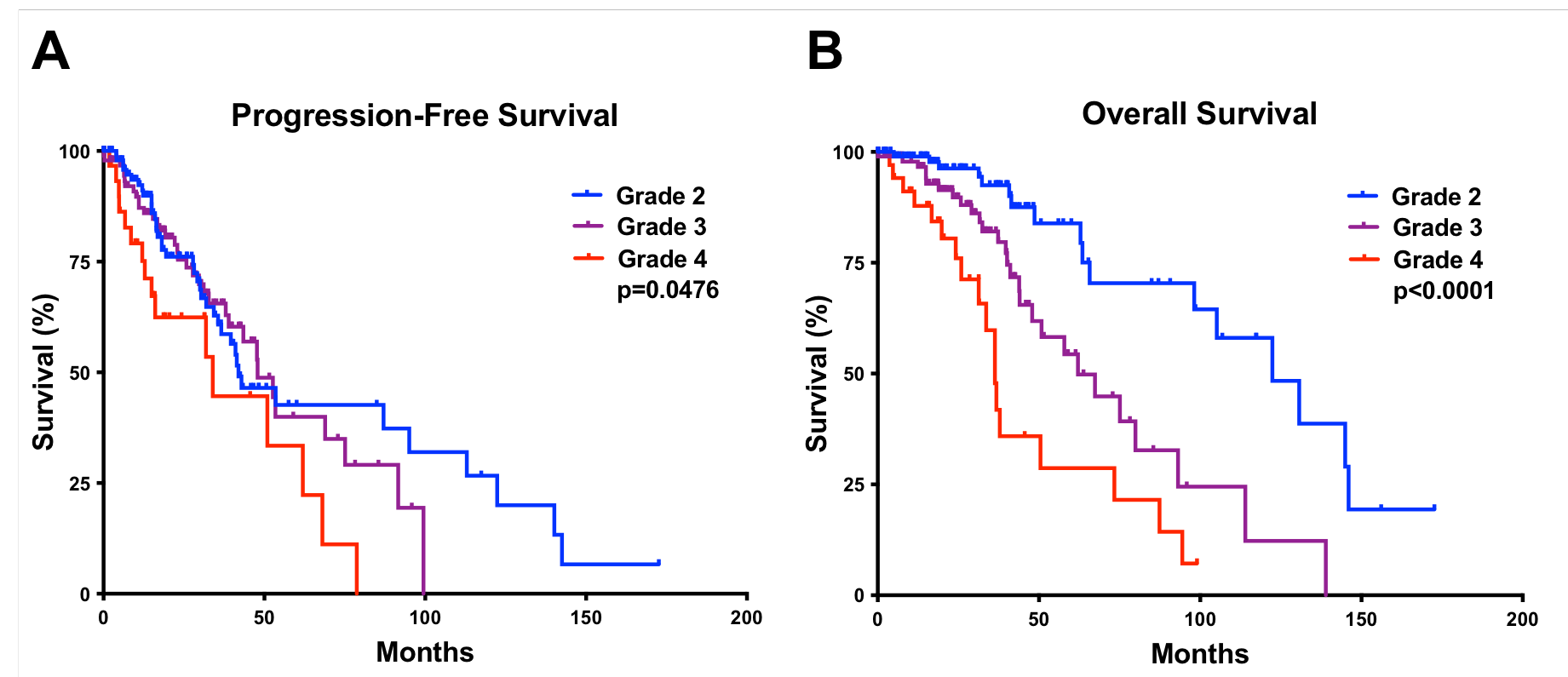

Supplement: Supplementary file 2 — Additional file 2. Fig. 2 Kaplan-Meier analysis of the full test cohort demonstrating significant differences in progression-free (A) and overall survival (B) between cases stratified by 2021 WHO grade [file 40478_2022_1339_MOESM2_ESM.tiff]
